# Effectiveness of Information and Communication Technology on Obesity in Childhood and Adolescence: Systematic Review and Meta-analysis

**DOI:** 10.2196/29003

**Published:** 2021-11-17

**Authors:** Jihyun Park, Mi-Jeong Park, Young-Gyun Seo

**Affiliations:** 1 Department of Family Medicine Hallym University Sacred Heart Hospital Anyang Republic of Korea

**Keywords:** ICT, eHealth, mHealth, weight loss, obesity, BMI, meta-analysis, randomized controlled trial, children, adolescents, mobile phone

## Abstract

**Background:**

Internet or mobile device use as a form of information and communication technology (ICT) can be more effective in weight loss and weight maintenance than traditional obesity interventions.

**Objective:**

The study aims to assess the effectiveness of child-centered ICT interventions on obesity-related outcomes.

**Methods:**

Articles were retrieved from the Cochrane Central Register of Controlled Trials, Embase, and PubMed web-based databases. We selected randomized controlled trials in which the participants were aged <18 years. The primary outcomes were BMI, body weight, BMI *z*-score, waist circumference, and percentage body fat.

**Results:**

In total, 10 of the initial 14,867 studies identified in the databases were selected according to the inclusion criteria. A total of 640 participants were included in the intervention group and 619 in the comparator group. Meta-analyses were conducted considering various subgroups (intervention type, comparator type, target participants, mean age, sex, BMI status, and follow-up period). Overall, ICT interventions demonstrated no significant effect on BMI, body weight, BMI *z*-score, waist circumference, and percentage body fat. Subgroup analyses revealed that the effect of the intervention was statistically significant for the following: *web* intervention (weighted mean difference [WMD]=−1.26 kg/m^2^, 95% CI −2.24 to −0.28), *lifestyle modification* comparator (WMD=−1.75, 95% CI −2.76 to −0.74), intervention involving both *boys and girls* (WMD=−1.30, 95% CI −2.14 to −0.46), and intervention involving *obesity only* (WMD=−1.92, 95% CI −3.75 to −0.09).

**Conclusions:**

The meta-analysis results for children with obesity who used the *web* intervention program confirmed significant effects on BMI reduction compared with *lifestyle modification*. Evidence from the meta-analysis identified internet technology as a useful tool for weight loss in children with obesity.

## Introduction

### Background

The prevalence of obesity among children and adolescents worldwide has increased at an alarming rate [1,2]. Children with obesity are now prone to developing diseases that were only observed in adults, including high blood pressure, impaired glucose tolerance, type 2 diabetes mellitus, dyslipidemia, sleep apnea, joint problems, and fatty liver disease [3-5]. In addition, children with obesity may be at risk for a variety of social and psychological problems such as low self-esteem, bullying, and discrimination [6].

Given the adverse health outcomes and high prevalence rate, effective interventions are imperative for the management of childhood obesity. Traditionally, many forms of intervention have been attempted to address childhood obesity, and they have mainly comprised lifestyle modifications and mental health care as well as medication and surgical treatment [7-10]. However, some methods have limited indications in children, and their effectiveness is debatable [11,12]. Therefore, it is necessary to develop more efficient and effective approaches for children.

### Information and Communication Technology

Internet and mobile use in childhood and adolescence are already becoming essential elements of young people’s lives [13-15], providing several advantages such as learning, information, and entertainment but also causing many problems. Excessive internet and mobile use has resulted in more sedentary behavior, decreased physical activity, and unhealthy dietary patterns, and it is emerging as a social problem that suffices diagnosis as a form of addiction disorder [16]. In addition, studies have reported that increased screen time and obesity are strongly correlated [17].

In contrast, there have been some attempts to use active internet and mobile use during childhood as a means of obesity intervention. Mobile health (mHealth), defined as *medical and public health practice supported by mobile devices, such as mobile phones, patient monitoring devices, personal digital assistants, and other wireless devices* [18], has the potential to influence a variety of health outcomes and has become a key trend in health service provision during recent years [19]. In addition, previous research has demonstrated that internet-based behavioral interventions have the potential for weight management [20-22].

Information and communication technology (ICT) is a technology that can be used to connect information technologies such as computers and software with communication technologies such as telephones and telecommunication networks. ICT includes cell phone calls, SMS, and apps using a mobile phone, email, and web services using a computer, and telehealth, including health education services, remote monitoring, and remote counseling [23].

### Objectives

Internet or mobile device use as a form of ICT can be more effective in weight loss and weight maintenance than traditional obesity intervention, as it offers benefits in terms of cost, ease of use, accessibility, and time to visit, while improving compliance with prescribed treatments through extensive patient monitoring and continuous support.

Therefore, we conducted a systematic review and meta-analysis to assess the effectiveness of child- and adolescent-centered ICT interventions on obesity-related outcomes.

## Methods

### Overview

We performed a meta-analysis based on the Cochrane Handbook for Systematic Reviews of Interventions [24] and the Centre for Reviews and Dissemination’s guidance for undertaking reviews in health care [25]. We reported based on the PRISMA (Preferred Reporting Items for Systematic Reviews and Meta-Analyses) statement [26].

### Literature Search

A systematic search of the effects of ICT on obesity-related outcomes was conducted. We searched the Cochrane Central Register of Controlled Trials, Embase, and PubMed web-based databases and retrieved articles published before January 1, 2021. The search terms used were as follows: *ICT OR information and communication technology OR Internet OR web OR social media OR mobile OR smartphone OR application OR app* AND *obesity OR obese OR weight OR metabolic syndrome*. The search was limited to randomized controlled trials (RCTs) and English articles; however, there were no restrictions on the calendar date. Reference lists of the retrieved articles were also reviewed. Information that was unavailable in the selected articles was requested by contacting the relevant authors; however, no response was received.

Two of the authors (JP and MJP) independently reviewed the titles and abstracts after the removal of duplicates. Discrepancies were resolved either by a discussion between the authors or by requesting comments from the third author (YGS). The 3 authors independently analyzed the full text of the remaining articles to determine the final inclusion.

### Eligibility Criteria

We selected the trials to be included in the meta-analysis using the following criteria: (1) the trial was a human RCT written in English, and the full text was available; (2) participants were aged <18 years; (3) the intervention group underwent ICT intervention alone or along with other lifestyle interventions; (4) the comparator group did not undergo ICT intervention; (5) the trial included an assessment of the following primary outcomes: BMI, body weight (BW), BMI *z*-score, waist circumference (WC), and percentage body fat (%BF); and (6) mean values of changes from baseline (or postintervention values if not available) with SD (or data suitable for calculating SD: 95% CI or SE). Uncontrolled, cross-sectional, and animal studies were excluded. The selection criteria did not limit the type of ICT used.

### Risk of Bias Assessment

We used guidelines from the Cochrane Handbook for Systematic Reviews of Interventions to assess the risk of bias in the RCTs [24]. Sources of bias, such as selection bias (random sequence generation and allocation concealment), detection bias (blinding of outcome assessment), attrition bias (incomplete outcome data), and reporting bias (selective reporting) were evaluated. Each domain was assessed in terms of methodological quality, with low or high risk of bias. If data were insufficient to make a reasonable judgment, the domain was described as *unclear risk of bias*.

The risk of bias was reported graphically using Review Manager (RevMan, Version 5.3; Copenhagen: The Nordic Cochrane Center, The Cochrane Collaboration, 2014).

### Data Extraction

Data were independently extracted by 2 authors (JP and YGS) from the selected RCTs. From each RCT, the following data were extracted: name of the first author, year of publication, country where the RCT was performed, sample size, participant-related variables (age, sex, and BMI status), intervention-related variables (ICT type, study duration, target participants, intervention details, comparator details, intervention frequency, and feedback frequency), and treatment effects (mean difference and SD of 2 time point values or mean and SD of postintervention values). The primary outcomes were BMI, BW, BMI *z*-score, WC, and %BF.

### Data Synthesis

The data set was constructed using the mean differences and SDs between the pre- and postintervention values. When the mean difference and SD were not published, the mean and SD of the postintervention values were used. In a meta-analysis, it was possible to combine both the mean differences and the means of postintervention values, assuming that the relative effects assessed by both the mean differences and the means of postintervention values are the same [24]. The final results were calculated and aggregated by one author (YGS).

### Meta-analysis

For the meta-analysis, we used Stata/MP (version 14.0; StataCorp). The weighted mean differences (WMDs) of BMI, BW, BMI *z*-score, WC, and %BF in the intervention and comparator groups were calculated. We used the Cochran Q test and I^2^ test to test the heterogeneity between the study results. For interpretation, I^2^ values of 25, 50, and 75 were considered to represent low, moderate, and high heterogeneity, respectively [27]. To consider heterogeneity, the DerSimonian and Laird [28] random-effects model for estimating WMD with 95% CI was used. The effect size and 95% CI of each study were expressed as forest plots. We checked the symmetry of the funnel plots to evaluate the presence of publication bias. In addition, we used the Egger regression test to evaluate the small study effects [29]. Heterogeneity between studies was analyzed using a meta-regression. We used covariates that may influence the association between ICT and BMI, namely, intervention type (web vs web plus vs app vs app plus), comparator type (control vs print-based vs lifestyle modification), target participants (parents and children vs children vs parents), mean age (<10 vs ≥10 years), sex (boys and girls vs boys vs girls), BMI status (normal to obese vs overweight or obesity vs obesity), and follow-up period (≥6 vs <6 months). The cutoff for the intervention period (6 months) was based on the transtheoretical model [30]. The statistical significance level was set at 5%. For heterogeneity, a threshold *P* value of .10 estimated using the Cochran Q test was considered statistically significant [27].

## Results

### Study Selection and Characteristics

**Figure 1:**
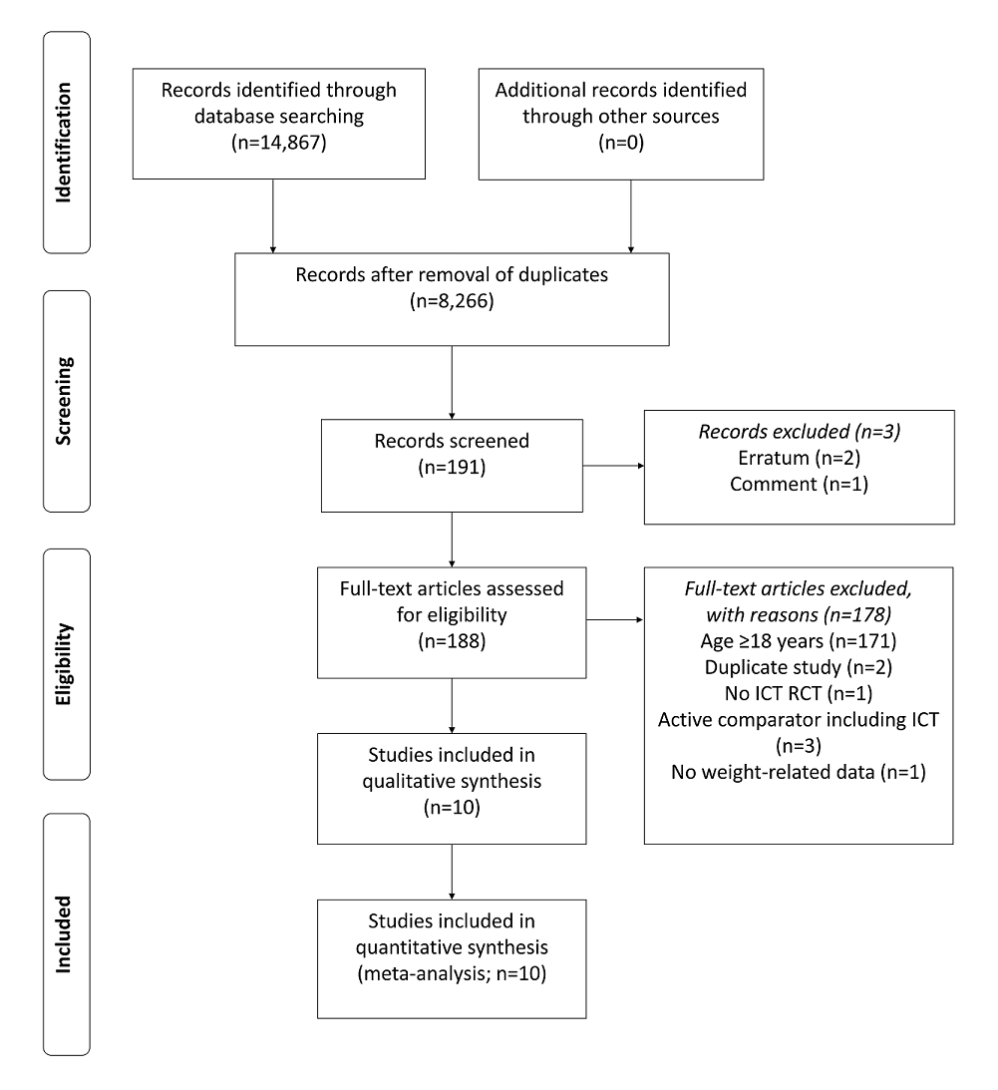
The PRISMA (Preferred Reporting Items for Systematic Reviews and Meta-Analyses) flow diagram for study selection. ICT: information and communication technology; RCT: randomized controlled trial.

In total, 10 [31-40] of the initial 14,867 studies identified in the databases were selected according to the inclusion criteria, and they contained sufficient data for meta-analysis (Figure 1). The meta-analysis included 13 data sets (2 studies each had 2 ICT types [32,34]: web and web plus; one study had two comparator types [38]: lifestyle modification and lifestyle modification plus). A total of 640 participants were included in the intervention group (range of the number of participants, 15-181) and 619 (range of the number of participants, 13-180) in the comparator group. All participants were aged <18 years. Of the included studies, one evaluated boys only [40], another evaluated girls only [39], and the other 8 did not differentiate between the sexes of the participants. Six out of 10 studies had intervention periods of 12 weeks, and the remaining 4 had intervention periods >12 weeks. Five studies had follow-up periods of 12 weeks, and the remaining 5 had follow-up periods of >12 weeks. The frequency of interventions and feedback varied from study to study. The average attendance rate of the participants in the study was 86.49% (1089/1259). The characteristics of the selected RCTs are summarized in Table 1.

**Table 1:** Characteristics of the selected randomized clinical trials.

Study (country)	Number at baseline→follow-up	Age, range or mean (SD)	Sex (% males)	BMI, range or mean (SD)	BMI status	ICT^a^ type	Intervention duration (weeks)	Target participants	Intervention details	Comparator details	Intervention frequency	Feedback frequency
Johansson et al [31] (Sweden)	IG^b^ 15→9; CG^c^ 13→9	5-12	Male or female (46.4)	Boys: BMI ≥98.9th percentile; girls: BMI ≥98.6th percentile	Obesity	App	24	Parent and children	Provement: display weight loss target curve, exchange text messages; Lifee Spirits+activity monitor: increase motivation for physical activity	LSM^d^ (improving dietary habits and increasing physical activity to reduce the degree of obesity)	Daily monitoring, messages whenever they felt a need for support	At least weekly
Chai et al [32] (Australia)	IG (telehealth) 16→11; IG (telehealth+SMS) 15→10; CG 15→15	4-11; 9 (2.3)	Male or female (59)	22.5 (5.1)	Overweight or obesity	Telehealth or telehealth+SMS	12	Parent and children	Telehealth dietitian consultation: semistructured telehealth consultations (approximately 20 minutes each); website (Back2Basics Family): information on various nutrition topic; Facebook group: exchange ideas and information related to the Back2Basics Family website; SMS to parents: targeting healthy eating for children	Waitlist control	Telehealth: week 1, 4; website: preferred time and frequency; Facebook: weekly; SMS: 4-weekly rotations of decreasing frequency (ie, 5, 4, 3, 2 per week)	Week 1, 4
Delisle Nyström et al [33] (Sweden)	IG 156→133; CG 159→130	4.5	Male or female (39)	IG: 15.9 (1.4); CG: 15.7 (1.2)	Normal to obesity	App	24	Parent	General information, advice, and evidence-based strategies on how to change unhealthy behaviors; register child’s intake of fruits, vegetables, candy, sweetened beverages, and sedentary time; submit questions to a dietician and a psychologist to ask questions specific to their child	A pamphlet on healthy eating and physical activity	At least weekly	Weekly
Bruñó et al [34] (Spain)	IG (Move It) 18→15; IG (Move It plus) 16→15; CG 18→13	9-16; 12.6 (1.7)	Male or female (57.7)	BMI ≥85th percentile	Overweight or obesity	Web or web+email	12	Children	Move It: web-based physical exercise program combines one aerobic exercise (brisk walking) and 10 muscular strength exercises; Move It plus: move It+weekly reminder and motivational emails	The same exercise program as the intervention group by a written guide	60 sessions distributed over 3 months, with 5 weekly sessions of 60 minutes each	Weekly
Rerksuppaphol and Rerksuppaphol [35] (Thailand)	IG 111→111; CG 107→106	10.7 (3.1)	Male or female (49)	8.36 kg/m^2^ (IQR 16.08-22.09 kg/m^2^)	Normal to obesity	Web	16	Children	Personal data collection, anthropometric variables and the interpretation of nutritional status, information related to healthy nutrition, food habits and physical activity by web	The same program as the intervention group by trained research assistants	Monthly	Monthly
Mameli et al [36] (Italy)	IG 23→16; CG 20→14	10-17	Male or female (61.9)	BMI ≥95th percentile	Obesity	App+wrist band	12	Children	App: measure energy intake; wrist band: measure energy expenditure; SMS: feedback	LSM (the Mediterranean diet and instruction to practice physical activity and minimize sedentary activity)	Data obtained by the wrist band and app were made available daily	Weekly
Mohammed Nawi and Che Jamaludin [37] (Malaysia)	IG 47→47; CG 50→50	16	Male or female (56.7)	BMI >25 kg/m^2^	Overweight or obesity	Web	12	Children	Information on healthy lifestyle, diet, and ways to overcome obesity, discussion by web	The same information as the intervention group by the pamphlets	Weigh and calculate BMI every 2 weeks; notified with any updates and information	Chat sessions on the website; monitoring negative comment by admin
Abraham et al [38] (China)	IG 16→16; CG (sLMP^e^) 16→16; CG (control) 16→16	12-18	Male or female (60.4)	BMI ≥95th percentile	Obesity	Web+cell phone calls+SMS	12	Children	Internet-based curriculum (nutrition and physical activity); cell phone follow-up; weekly semipersonalized SMS	Control: usual care consisted of a focused dietary and physical activity history, medical history, physical examination, laboratory screening and obesity counseling; sLMP: usual care+four meetings with a nutritionist over 3 months	Goal setting: monthly	Weekly SMS
Nollen et al [39] (United States)	IG 26→22; CG 25→22	9-14; 11.3 (1.6)	Female (0)	23.7 (5.7)	Normal to obesity	Standalone mobile app	12	Children	Set 2 daily goals and an accompanying plan for improving the behavior, and feedback and reinforcement on goal-attainment	The same contents as the intervention group by the manuals	Self-monitor progress toward their goals at 5 times a day	Five times a day
Smith et al [40] (Australia)	IG 181→139; CG 180→154	12-14; 12.7 (0.5)	Male (100)	20.5 (4.1)	Normal to obesity	App	20	Children	Supplement the delivery of enhanced school sport and interactive sessions by providing participants with physical activity monitoring, recording of fitness challenge results, tailored motivational messaging, peer assessment of resistance training skills, and goal setting for physical activity and screen-time	Participate in usual practice (regularly scheduled school sports and physical education lessons)	Tailored motivational and informational *push prompt* messages	Peer assessment

^a^ICT: information and communication technology.

^b^IG: intervention group.

^c^CG: comparator group.

^d^LSM: lifestyle modification.

^e^sLMP: simplified lifestyle modification program.

### ICT Interventions

Of the included 10 studies, 5 (50%) were web-based interventions, and the other 5 (50%) were app-based interventions.

Two of the 5 web-based intervention studies were *web* interventions, one for providing web-based nutrition and physical activity information for children [35] and one for providing web-based nutrition information for children [37]. One of the 5 web-based intervention studies was *web* or *web plus* intervention, which provided web-based physical exercise programs and motivational emails for children [34]. Two of the 5 web-based intervention studies were *web plus* interventions, one for providing web-based nutrition and physical activity information as well as SMS feedback for children [38] and one for providing web-based telehealth dietitian consultation, nutrition information, and SMS feedback for parents and children [32].

Four of the 5 app-based intervention studies were *app* interventions, one for providing app-based weight loss target curve, physical activity information for parents and children [31], one for providing app-based nutrition information for parents [33], one for providing app-based nutrition and screen-time information for children [39], and one for providing app-based physical activity and screen-time information for children [40]. One of the 5 app-based intervention studies was *app plus* intervention, which provided app-based nutrition and physical activity information and SMS feedback for children [36].

### Risk of Bias

Participant blinding was not possible because of the characteristics of the intervention. Therefore, performance bias was not considered in the risk of bias assessment. There was some risk of bias in the individual studies. Two studies lacked sufficient data to evaluate the randomization sequence generation. Four studies lacked sufficient data to evaluate allocation concealment. Three studies indicated blinding of outcome assessment; however, one study stated that assessors were not blinded at follow-up. Two studies lacked sufficient data to evaluate the attrition bias. Three studies lacked sufficient information to evaluate the study protocol, and one study did not report all of the information. The risk of bias assessment is reported graphically in Figure S1 in Multimedia Appendix 1 [31-40].

### Synthesis of Results

**Figure 2:**
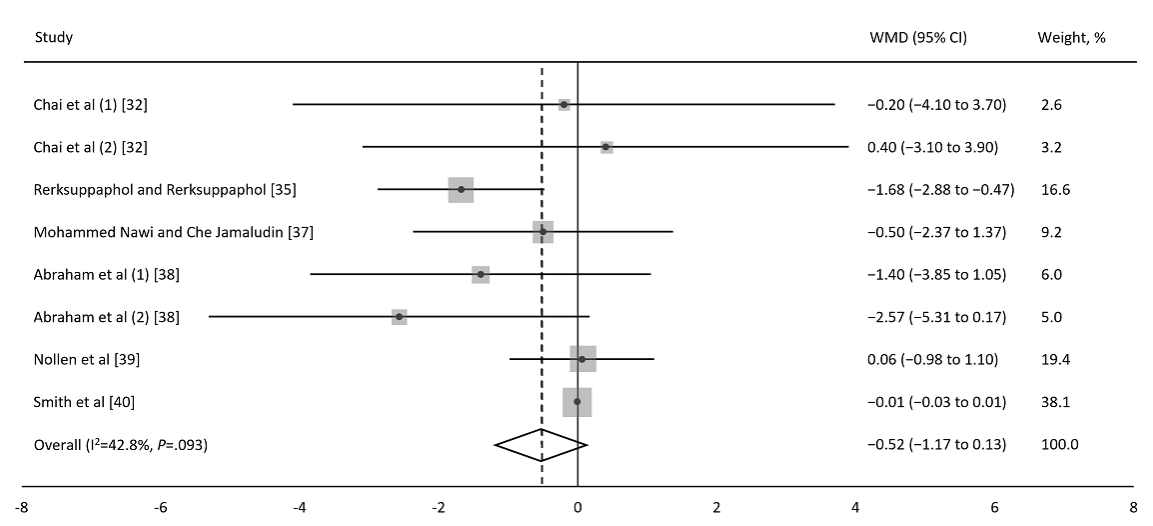
Forest plot for changes in BMI. Meta-analysis of the effect of the information and communication technology (ICT) on BMI (kg/m^2^). The mean difference for each study reporting changes in BMI is depicted along with the 95% CI. The random-effects model was used to estimate the weighted mean differences with 95% CIs. Negative values favor ICT because the ICT intervention group experienced more BMI reduction than the comparator group did. WMD: weighted mean difference.

To evaluate the overall intervention effect, we calculated the mean difference in BMI for each study. Figure 2 shows the effect size for each study and the overall effect size. The intervention demonstrated no significant effect on BMI (WMD=−0.52 kg/m^2^, 95% CI −1.17 to 0.13). Categorization of target participants, mean age, and BMI appeared to have moderate to high heterogeneity. Categorization of the follow-up period appeared to have low to moderate heterogeneity. For all other categories, the heterogeneity was low. We also calculated mean differences in BW, BMI *z*-score, WC, and %BF to determine the overall intervention effect, and no significant intervention effects were identified for BW (WMD=−0.22 kg, 95% CI −1.05 to 0.62), BMI *z*-score (WMD=−0.22, 95% CI −0.49 to 0.04), WC (WMD=−1.70 cm, 95% CI −3.91 to 0.51), and %BF (WMD=−0.00%, 95% CI −0.07 to 0.07; Figure S2 in Multimedia Appendix 1).

### Publication Bias

**Figure 3:**
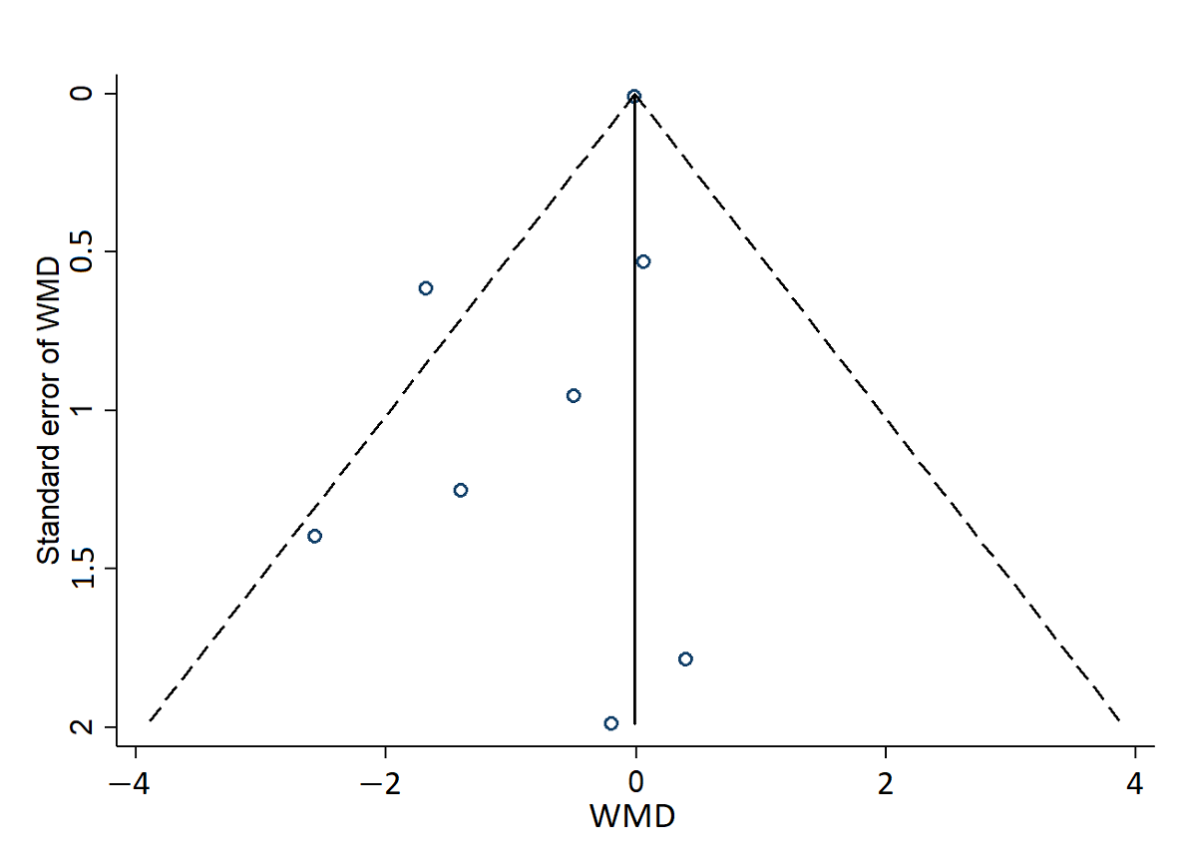
Funnel plot for changes in BMI. The funnel plots of SE of weighted mean difference (WMD) against WMD for BMI to assess for publication bias. WMD: weighted mean difference.

To verify possible publication bias, we plotted the effect size against the SE to generate a funnel plot (Figure 3). There was no statistically significant publication bias according to the Egger test (*P*=.09).

### Meta-regression

The results of the simple meta-regression analysis were significant for categorical covariates of intervention type (β=.69, 95% CI 0.10 to 1.28) and comparator type (β=−.73, 95% CI −1.39 to −0.07). A subgroup analysis by intervention type revealed that the intervention effect was statistically significant only in the *web* intervention (WMD=−1.26 kg/m^2^, 95% CI −2.24 to −0.28). A subgroup analysis by comparator type demonstrated that the intervention effect was statistically significant only in the *lifestyle modification* comparator (WMD=−1.75 kg/m^2^, 95% CI −2.76 to −0.74). A subgroup analysis by sex revealed that the intervention effect was statistically significant only in the intervention involving both *boys and girls* (WMD=−1.30 kg/m^2^, 95% CI −2.14 to −0.46). In addition, a subgroup analysis by BMI status demonstrated that the intervention effect was statistically significant only in the intervention involving *obesity only* (WMD=−1.92 kg/m^2^, 95% CI −3.75 to −0.09; Figure S3 in Multimedia Appendix 1).

Subgroup analyses were also performed for other outcome variables, and the results of subgroups containing only one study data were excluded. For BW, subgroup analysis revealed that the intervention effect was statistically significant only in the *web* intervention (WMD=−1.21 kg, 95% CI −2.36 to −0.06) and the intervention involving a follow-up period *<6 months* (WMD=−0.87 kg, 95% CI −1.73 to −0.01; Figure S4 in Multimedia Appendix 1). For BMI *z*-score, subgroup analysis demonstrated that the intervention effect was statistically significant only in the intervention targeting *parents and children* (WMD=−0.23, 95% CI −0.37 to −0.08) and that where the average age of participants was *<10 years* (WMD=−0.23, 95% CI −0.37 to −0.08; Figure S5 in Multimedia Appendix 1). For WC, subgroup analysis revealed that the intervention effect was statistically significant only in the *web plus* intervention (WMD=−4.88 cm, 95% CI −8.93 to −0.83), *lifestyle modification* and *control* comparator (WMD=−3.58 cm, 95% CI −6.30 to −0.85 and WMD=0.50 cm, 95% CI 0.43 to 0.57, respectively), intervention involving both *boys and girls* (WMD=−2.95 cm, 95% CI −5.18 to −0.71), and intervention involving *obesity only* (WMD=−5.50 cm, 95% CI −9.89 to −1.12; Figure S6 in Multimedia Appendix 1). Subgroup analyses demonstrated no statistically significant effect on %BF (Figure S7 in Multimedia Appendix 1).

### Behavioral Change

Nine of the included 10 studies reported the initial results, and only one study reported the results of an additional 6 months follow-up after reporting the initial results of a 6-month intervention [33].

Five of the included 10 studies also reported results on behavioral changes. Some studies have shown that there were no significant intervention effects on sedentary time [33], physical activity [33,38], consumption of fruits and vegetables, and sugar-sweetened beverages [33,39]. However, one study reported that screen-time and sugar-sweetened beverage consumption [40] were improved, and another study reported that dietary intake was improved in the intervention group [32]. One study reported no significant intervention effects on stress or dietary knowledge scores [38]. However, another study reported that emotional functioning significantly increased after the intervention [37].

## Discussion

### Principal Findings

This study identified the effects of ICT intervention on obesity-related outcomes in children and adolescents in RCTs through a systematic literature review and meta-analysis. We found that although ICT intervention is not significantly effective in reducing BMI, BW, BMI z-score, WC, and %BF, it does work in certain groups.

A subgroup analysis by intervention type revealed that the intervention effect was statistically significant only in the *we*b or *web plus* intervention for BMI, BW, and WC. This result is consistent with those of previous studies that showed that web-based health programs are effective in managing obesity. Meta-analyses have demonstrated that web-based interventions are effective in achieving weight loss in adults [41,42]. Several systematic reviews have explored the use of web-based interventions for the prevention or treatment of obesity and related conditions in pediatric populations [20,22]. In addition, a meta-analysis demonstrated that mobile-based interventions are effective in achieving weight loss [43,44] and reducing BMI [45-47] in adults. However, there has been no meta-analysis of mobile-based obesity intervention studies in children. Several systematic reviews have indicated that mobile-based interventions in obesity treatment programs have a modest effect on weight control [47-51]. However, these effects are inconsistent. Owing to the nature of mobile use, access is possible from anywhere; hence, the possibility of giving formal responses in situations where participants are unprepared to take certain actions cannot be ruled out. Therefore, additional RCTs and meta-analyses targeting the weight-loss effect of mobile-based interventions in children and adolescents are warranted.

Another subgroup analysis by comparator type revealed that the intervention effect was significant only in the *lifestyle modification* comparator rather than in the *control* comparator for BMI and WC. A previous meta-analysis [52] that analyzed the weight loss effect of circuit training demonstrated that focusing on circuit training alone rather than adding other lifestyle interventions to circuit training is effective. Among the RCTs included in our meta-analysis, 2 [35,38] had the *lifestyle modification* comparator. In these 2 studies, the comparator group did not focus on one lifestyle, intervened in various lifestyle habits, and the number of contacts was not frequent. Recognizing that they are undergoing an intervention, participants tend to act passively, hoping to elicit changes to their lifestyle habits; therefore, where there is no intensive intervention, the intervention can be counterproductive. However, as there may be other factors influencing the research results, additional studies comparing ICT with a comparator group focusing on one lifestyle are needed.

For WC, a significant intervention effect was also obtained when the comparator type was *control.* Among the RCTs included in our meta-analysis, 2 [32,40] had the *control* comparator. However, the difference in sample size was large between the 2 studies; thus, the results of the subgroup were not different from those of one study [40], which had a large sample size. Therefore, it is unreasonable to interpret this as a subgroup result.

The intervention effect was significant only in the intervention involving both *boys and girls* for BMI and WC. Among the RCTs included in our meta-analysis, one evaluated boys only [40], another evaluated girls only [39], and the other 8 did not differentiate between the sexes of the participants. Sex differences in response to ICT interventions are likely to be because of differences in participation. In cases where boys and girls participate together, the resultant mutual competition can increase participation, which subsequently increases the effect of the intervention. Several studies have compared the sex-dependent effects of school-based physical activity interventions [53-56]. However, to date, no meta-analysis has shown sex differences in the effects of ICT intervention on children with obesity. Therefore, additional RCTs are needed to investigate the sex-dependent weight loss effects of ICT interventions.

In addition, the intervention effect was significant only in the intervention involving obesity only for BMI and WC. Participants with normal weight or overweight status may be less motivated than with participants with obesity, which may dilute the overall effect. It is encouraging to identify meaningful results from studies solely involving children and adolescents with obesity. In other words, significant results can be obtained if ICT intervention is implemented in children and adolescents with obesity. Further well-designed ICT intervention studies targeting children and adolescents with obesity should be conducted.

When both parents and children were involved in children less than 10 years of age, a reduction in BMI *z*-score was observed. In the same situation, other outcome variables did not show a statistically significant effect; however, considering the BMI *z*-score only, ICT could be used as a means of preventing obesity-related outcomes by intervening in early childhood with the involvement of parents. Therefore, there is a need for additional long-term RCTs involving high-quality ICT interventions targeting parents and children under 10 years of age.

Previous studies have demonstrated that mHealth programs or internet technology have a higher attrition rate than conventional face-to-face methods [57,58]. However, the average attendance rate of the participants in this meta-analysis was 86.5%. Children with obesity are often ashamed because of social stigma and may face prejudice against weight [59]. Moreover, because children and teenagers have a high level of interest in and concentration with electronic devices, access through mobile means or the internet can be more efficient [60]. Therefore, the effect of obesity intervention through mHealth programs or internet technology is considered superior because of enhanced accessibility and an increased participation rate compared with conventional interventions such as the face-to-face method.

The advantage of childhood-obesity management using mHealth programs or internet technology is that it is possible to operate programs led by peer participants and provide real-time feedback. In particular, by presenting a mission aimed at improving dietary habits or increasing exercise among peers as well as providing rankings or rewards, it is possible to induce mutual participation through goodwill competition. However, among the studies included in this study, no study was conducted in a manner that induced competition among participants. Therefore, further research on the participation rates is imperative.

### Strengths

Our meta-analysis had the following strengths: First, to the best of our knowledge, this is the first meta-analysis to assess the association between ICT intervention and weight loss in children and adolescents. Second, we examined the differences in weight-loss effects among subgroups according to the type of intervention, type of comparator, target participants, mean age, sex, BMI status, and follow-up period. We found that ICT intervention is effective for weight loss in the *web* intervention, *lifestyle modification* comparator, intervention involving both *boys and girls*, and intervention involving *obesity only*. Finally, the heterogeneity among the included RCTs was low.

### Limitations

Our meta-analysis has several limitations. Although the meta-analysis found a moderate effect size that was statistically significant, as not many studies were included in the meta-analysis, generalization of the study results is limited because of potential publication bias. However, there were no small study effects according to the Egger regression test results. In addition, many of the studies were of short duration, making it unclear whether weight loss was sustained in the long term. Although the clinically significant threshold for weight loss was not always achieved across the studies, studies of longer duration might have found clinically significant weight loss. Therefore, further evidence is necessary to confirm this hypothesis. Outcome measurements based on participants’ self-reports for many studies were recorded using mobile apps and websites and the lack of comments on the reliability of the measurement method using mobile apps can limit the results analysis. In addition, no RCT has focused on sex differences in the use of mHealth programs and internet technology. Therefore, future research should investigate the sex-dependent weight loss effects of ICT interventions.

### Conclusions

The meta-analysis results for children and adolescents with obesity who participated in the *web* intervention program confirmed significant effects on BMI reduction compared with the *lifestyle modification* intervention. Evidence from the meta-analysis identified internet technology as a useful tool for weight loss in children and adolescents with obesity.

## Appendix

Multimedia Appendix 1 Risk of bias assessment and forest plots.

## Abbreviations

%BF: percentage body fat
BW: body weight
ICT: information and communication technology
mHealth: mobile health
PRISMA: Preferred Reporting Items for Systematic Reviews and Meta-Analyses
RCT: randomized controlled trial
WC: waist circumference
WMD: weighted mean difference
