# Supplementary material for: Effectiveness of Information and Communication Technology on Obesity in Childhood and Adolescence: Systematic Review and Meta-analysis
Source: J Med Internet Res. 2021 Nov 17;23(11):e29003. doi: 10.2196/29003 (PMC8726568; doi:10.2196/29003)
Supplement: Multimedia Appendix 1 [file jmir_v23i11e29003_app1.docx]

**Figure S1.** Risk of bias identified in the studies included in the meta-analysis

**(a) Risk of bias graph**

**
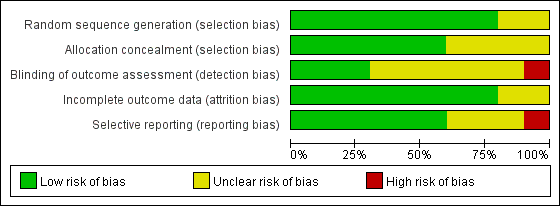
**

**(b) Risk of bias summary**


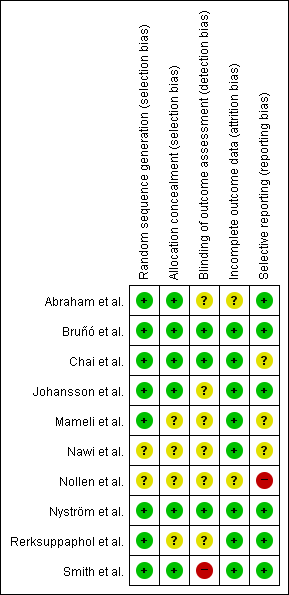


**Figure S2.** Forest plot for changes in other outcomes

Meta-analysis of the effect of the information and communication technology on body weight (kg), body mass index z-score, waist circumference (cm), and percentage body fat (%). The mean differences for each study reporting changes in body weight, body mass index z-score, waist circumference, and percentage body fat are depicted along with the 95% confidence interval (CI). The random-effects model was used to estimate the weighted mean differences (WMDs) with 95% CIs. Negative values favor information and communication technology because the information and communication technology intervention group experienced more body weight, body mass index z-score, waist circumference, or percentage body fat reduction than the comparator group did.

WMD, weighted mean difference; CI, confidence interval

(a) body weight

(b) body mass index z-score

(c) waist circumference

(d) percentage body fat

**Figure S3.** Forest plot for changes in body mass index according to subgroups

Meta-analysis of the effect of the information and communication technology on body mass index (kg/m^2^). The mean difference for each study reporting changes in body mass index is depicted along with the 95% confidence interval (CI). The random-effects model was used to estimate the weighted mean differences (WMDs) with 95% CIs. Negative values favor information and communication technology because the information and communication technology intervention group experienced more body mass index reduction than the comparator group did.

WMD, weighted mean difference; CI, confidence interval

(a) intervention type

(b) comparator type

(c) target participants

(d) mean age

(e) sex

(f) body mass index status

(g) follow-up period

**Figure S4.** Forest plot for changes in body weight according to subgroups

Meta-analysis of the effect of the information and communication technology on body weight (kg). The mean difference for each study reporting changes in body weight is depicted along with the 95% confidence interval (CI). The random-effects model was used to estimate the weighted mean differences (WMDs) with 95% CIs. Negative values favor information and communication technology because the information and communication technology intervention group experienced more body weight reduction than the comparator group did.

WMD, weighted mean difference; CI, confidence interval

(a) intervention type

(b) comparator type

(c) target participants

(d) mean age

(e) sex

(f) body mass index status

(g) follow-up period

**Figure S5.** Forest plot for changes in body mass index z-score according to subgroups

Meta-analysis of the effect of the information and communication technology on body mass index z-score. The mean difference for each study reporting changes in body mass index z-score is depicted along with the 95% confidence interval (CI). The random-effects model was used to estimate the weighted mean differences (WMDs) with 95% CIs. Negative values favor information and communication technology because the information and communication technology intervention group experienced more body mass index z-score reduction than the comparator group did.

WMD, weighted mean difference; CI, confidence interval

(a) intervention type

(b) comparator type

(c) target participants

(d) mean age

(e) sex

(f) body mass index status

(g) follow-up period

**Figure S6.** Forest plot for changes in waist circumference according to subgroups

Meta-analysis of the effect of the information and communication technology on waist circumference (cm). The mean difference for each study reporting changes in waist circumference is depicted along with the 95% confidence interval (CI). The random-effects model was used to estimate the weighted mean differences (WMDs) with 95% CIs. Negative values favor information and communication technology because the information and communication technology intervention group experienced more waist circumference reduction than the comparator group did.

WMD, weighted mean difference; CI, confidence interval

(a) intervention type

(b) comparator type

(c) target participants

(d) mean age

(e) sex

(f) body mass index status

(g) follow-up period

**Figure S7.** Forest plot for changes in percentage body fat according to subgroups

Meta-analysis of the effect of the information and communication technology on percentage body fat (%). The mean difference for each study reporting changes in percentage body fat is depicted along with the 95% confidence interval (CI). The random-effects model was used to estimate the weighted mean differences (WMDs) with 95% CIs. Negative values favor information and communication technology because the information and communication technology intervention group experienced more percentage body fat reduction than the comparator group did.

WMD, weighted mean difference; CI, confidence interval

(a) intervention type

(b) comparator type

(c) target participants

(d) mean age

(e) sex

(f) body mass index status

(g) follow-up period
